# Tunable Active Wien Filters Based on Memristors

**DOI:** 10.3390/mi16070769

**Published:** 2025-06-30

**Authors:** Elena Solovyeva, Artyom Serdyuk, Yury Inshakov

**Affiliations:** Department of Electrical Engineering Theory, Saint Petersburg Electrotechnical University “LETI”, 197022 St. Petersburg, Russia; serduk_artem_99@mail.ru (A.S.); inshakov40@mail.ru (Y.I.)

**Keywords:** memristive device, memristor, filter, tunable characteristic, modeling, LTspice

## Abstract

Devices with tunable characteristics and parameters are used in many technical fields. Such devices can be based on memristors, which serve as programmable potentiometers. The quality of the tuning is higher by means of memristors than with mechanical and digital potentiometers. We investigate a bandpass filter in the form of an active Wien bridge with a memristor. The filter is analyzed with the help of the nodal voltage method. The dependence of the resonance frequency on the parameters of the Wien circuit, the dependence of the quality factor, and the filter gain at resonant frequency on the parameters of the voltage divider are obtained. The dependences of the resonant frequency, quality factor, and gain at the resonant frequency on the parameters of the Wien filter were formed. The tuning of the main frequency features (the filter gain, quality factor, and resonance frequency) is shown to be independent. Under different values of memristance, the frequency features result from a simulation in LTspice. These features are less than 1 percent different from the corresponding features obtained analytically. Thus, the high precision of modeling and tuning of the frequency characteristics of the memristive Wien filter is demonstrated.

## 1. Introduction

When designing electronic equipment in the fields of radio engineering, electrical engineering, robotics, acoustics, biomedicine, control systems, etc., there is a need to develop devices with tunable parameters and characteristics, among which filters play a significant role. Filters are used for signal selection in monitoring, measurement, information, control, acoustic, and hydroacoustic systems; in radio–electronic equipment for correcting communication channel characteristics to improve their performance; in biomedical equipment for diagnostics and physiological research, etc. [1,2,3]. Based on the signal processing, filters are divided into linear and nonlinear, and analog and digital. This diversity of filters reflects the variety of tasks they address, such as nonlinear filtering of external non-Gaussian noise [4,5,6,7]; compensation of nonlinear signal distortions in devices [8,9,10,11,12,13]; removal of Gaussian noise [5,14,15]; extraction of useful signal spectra [16,17,18,19,20,21,22], etc.

Among analog selective filters, bandpass filters are widely used [16,17,18,19,23,24,25,26,27,28,29]. These filters are intended, for example, to ensure:

- Reliable signal transmission while suppressing noise that distorts useful signals
- Reliable operation of electrical equipment while minimizing interruptions, reducing losses, and improving the performance of electrical networks
- Protection of equipment from damage and prevention of failure risks by filtering interference and fault signals
- Maintaining stable operation of power supplies

The key requirements for tunable bandpass filters are the following:

- Minimal number of tunable elements
- Independent tuning of the filter’s main features (for instance, gain, quality factor, and resonant frequency)
- Low sensitivity of the transfer functions to deviations from the assigned parameters of circuit elements

**Figure 1:**
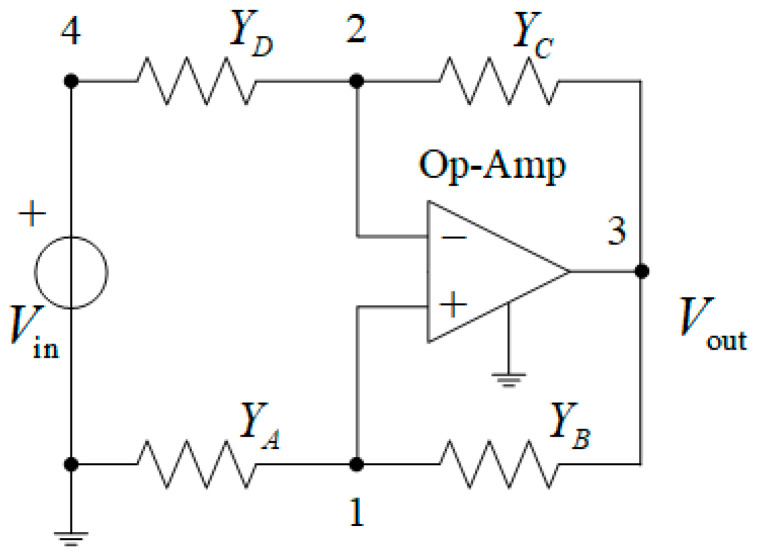
The circuit for the active bandpass Wien filter family.

The tunable bandpass active Wien filter, based on the Wien bridge with an operational amplifier (Op-Amp), meets the mentioned requirements. The family of active bandpass Wien filters is built on the circuit shown in Figure 1 [30]. The inclusion of an Op-Amp in the Wien bridge, with a frequency-dependent Wien circuit in the one arm and a voltage divider in the other, leads to the design of either an active filter or a sinusoidal oscillator, depending on whether the circuit is connected to the inverting or non-inverting input of the Op-Amp. To synthesize an active filter, the Wien circuit is connected to the inverting input of the Op-Amp, while the resistive voltage divider is connected to the non-inverting input of the Op-Amp [23,24,25,26,27,28].

**Figure 2:**
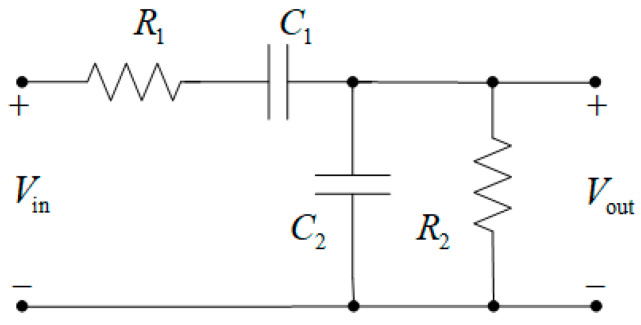
The frequency-dependent Wien circuit.

The frequency-dependent Wien circuit is shown in Figure 2. This circuit is a series connection of a high-pass filter (with elements *C*_1_ and *R*_1_) and a low-pass filter (with elements *C*_2_ and *R*_2_). As a result, a bandpass filter with a maximum gain at the resonant frequency is formed. At low frequencies, the reactance of the serial capacitor *C*_1_ is very high, so it acts as an open circuit, blocking any input signal *V*_in_, and thus resulting in practically no output signal *V*_out_. At high frequencies, the reactance of the parallel capacitor *C*_2_ becomes very low, so the parallel-connected capacitor acts as a short circuit at the output, therefore, causing the output signal to be small again [23,24,25,26,27,28].

The use of an Op-Amp in the Wien bridge allows for a large slope of amplitude–frequency response within a narrow frequency range, thus making the filter more selective by increasing its order. To achieve the desired characteristics, conventional passive filters typically contain two reactive elements (a capacitor and an inductor). The use of an Op-Amp enables the creation of high-order filters without inductors. This leads to more compact and cost-effective designs that can be implemented on integrated circuits. Additionally, due to the high input and low output impedance of operational amplifiers, cascading filter connections can be used to form the desired device characteristics [23,24,25,26,27,28].

Tuning the resonance frequency of the active Wien bridge is performed by adjusting resistances and capacitances in the Wien circuit. If the tuned parameters do not provide a sufficient signal level at the resonant frequency, fine tuning at this frequency can be accomplished by adjusting resistance in the voltage divider. Thus, it is possible to separately tune parameters of the active Wien filter (resonance frequency, gain at resonant frequency, and quality factor). The above can be used to optimize the filter performance by setting the desired bandwidth range and the level of signal suppression outside the bandwidth.

Tuning of the resistors in the active Wien filter can be performed using mechanical or digital potentiometers, which are included in the circuit shown in Figure 1. However, the use of mechanical potentiometers is challenging due to their low stability and discrete resistance adjustment. These factors make it difficult to implement automatic quality factor control of analog filters. Digital potentiometers also have a drawback: Their switches (usually MOSFET-based) introduce parasitic capacitance and resistance. This parasitic capacitance limits resolution and bit depth of the switched resistors, causes a problem with long-term charge storage, and reduces the reliability of the device [31,32].

A promising method for tuning parameters and characteristics of electronic devices (for instance, active Wien filters) is based on programmable memristor potentiometers. Such potentiometers ensure high precision in setting resistance values, and these values are programmed through pulse signals by changing their number, amplitude, frequency, and duty factor [32,33,34,35,36,37]. Thus, having high requirements for the accuracy of tunable parameters and characteristics, the use of memristors appears to be convenient and promising.

The advantages of memristor potentiometers derive from the advantages of memristors, which they are built on. The memristor is a resistor with memory. It “remembers” the resistance set by the applied voltage and does not change until the voltage is altered. Memristors have several advantages; the key ones include long-term information storage; energy efficiency (they operate at low voltages and consume little energy); small size (nanoscale), which enables high integration of memristors in circuits; multilevel memristance and its programmability (controllability); high switching speed; and compatibility with CMOS technology [38,39,40,41,42,43,44]. Due to these properties, memristors are used in many devices across various scientific and technical fields. Below are some of such devices [38,39,40,41,42,43,44,45]:

- Circuits with tunable characteristics. In many analog devices, such as amplifiers, attenuators, and filters, it is necessary to program resistors in order to change operating modes and compensate for parasitic noise. Programmable resistors with high resolution and the ability to correct for small parasitic interference are useful in many analog circuits.
- Nonlinear converters (oscillators, comparators, rectifiers, compensators, etc.).
- Various types of neural networks (multilayer feedforward, radial, recurrent, cellular, convolutional, neuro-fuzzy, etc.). These networks perform tasks such as modeling, filtering, controlling, signal generating, and speech and image processing in addition to ensuring hardware security and constructing associative memory. For instance, convolutional neural networks (CNNs) are developed through the implementation of long-term potentiation (LTP) and long-term depression (LTD) mechanisms. The LTP/LTD characteristics are realized on the basis of memristors. These mechanisms help to update weights of the CNN when optimizing the model. The CNNs with the memristor’s LTP/LTD characteristics are used for motion deblurring of license plate images and restoration enhancement of image clarity and readability [46]. In addition, a spiking neural network with neurons, which are built on proton-activated memristors, is used as a biomimetic humidity sensor for respiratory monitoring to diagnose lung disease [47].
- Neuromorphic systems. A neuromorphic system is a mixed-mode analog–digital system that mimics neural architecture by computing, modeling, and simulating the nervous system in real time. It is desirable for complex neural networks to have low power consumption; therefore, it is reasonable to use energy-efficient memristors, which form the basis of synapses—multipliers of signals by weight coefficients in neurons.
- Non-volatile memory devices. Resistive random-access memory (RRAM) is a device with the switching medium placed between the top and bottom electrodes. The resistance of the switching medium is controlled by an electrical signal (current or voltage) applied to the electrodes. In addition to RRAM, electrically non-volatile resistance changes are observed in ferroelectric RAM (FeRAM), magnetic RAM (MRAM), and phase-change RAM (PRAM). The memristor with a metal–insulator–metal structure exhibits resistive switching and is often used as a non-volatile memory.
- Programmable logic systems. Memristors are used for implication (the “If–then” logical operation) to perform all basic Boolean operations on two variables.

This paper consists of four sections.

Section 1 emphasizes the importance of tuning parameters and characteristics of various devices in many technical fields. Among such devices, a special place is given to filters due to their diverse functions: noise and fault signal suppression, distortion removal, spectral selectivity of processed signals, etc. This section examines the family of active bandpass Wien filters based on the Wien circuit, voltage divider, and Op-Amp. The key advantage of the filter is highlighted: the ability to independently change the resonance frequency, quality factor, and gain at resonant frequency. These are memristors to be proposed for parameter tuning, since their advantages provide superior performance compared to mechanical and digital potentiometers.

Section 2 presents an analysis of the active memristive Wien filter using the nodal voltage method. The analysis results in the derivation of the Wien filter’s transfer function, dependence of the resonant frequency on the Wien circuit parameters, and dependencies of the quality factor and gain at the resonant frequency on the voltage divider parameters for given Wien circuit parameters.

Section 3 provides the results of the circuit simulation of the active memristive Wien filter in LTspice and compares them with the results obtained through analytical calculations based on the nodal voltage method in Section 2.

Section 4 presents conclusions of the studies performed.

## 2. Analysis of the Active Memristive Wien Filter

The system of equations, composed using the nodal voltage method in the Laplace transform domain for the active Wien bridge circuit shown in Figure 1, is written as

(1) $$\left\{\begin{array}{l} (Y_{a}+Y_{b})V_{1}-0V_{2}-Y_{b}V_{3}-0V_{4}=0,\\ -0V_{1}+(Y_{d}+Y_{c})V_{2}-Y_{c}V_{3}-Y_{d}V_{4}=0,\\ V_{4}=V_{\mathrm{in}}, \end{array}\right.$$

where $Y_{a}$,…, $Y_{d}$ are the operator conductivities (in the Laplace transform domain) of the corresponding elements in Figure 1; $V_{1}$,…, $V_{4}$ are the voltages at corresponding nodes of the circuit relative to the reference node (grounded node, whose potential equals 0); and $V_{\mathrm{in}}$ is the input voltage of the circuit.

Based on the property of the ideal Op-Amp, we write $V_{2}=V_{1}$. The nodal equation for the third node is not formulated. Let us find the solution to the system of Equation (1), i.e.,

$$\left\{\begin{array}{l} (Y_{a}+Y_{b})V_{1}-Y_{b}V_{3}=0,\\ (Y_{d}+Y_{c})V_{1}-Y_{c}V_{3}=Y_{d}V_{\mathrm{in}}. \end{array}\right.$$

The obtained solution determines nodal voltages:

$$V_{1}=\frac{Y_{b}}{Y_{a}+Y_{b}}V_{3},$$

$$V_{3}=Y_{d}V_{\mathrm{in}}/\left[(Y_{d}+Y_{c})\frac{Y_{b}}{Y_{a}+Y_{b}}-Y_{c}\right].$$

The transfer function, expressed in terms of the nodal voltages, is as follows:

(2) $$H(s)=\frac{V_{3}}{V_{\mathrm{in}}}=\frac{V_{\mathrm{out}}}{V_{\mathrm{in}}}=\frac{Y_{d}(Y_{a}+Y_{b})}{Y_{b}(Y_{d}+Y_{c})-Y_{c}(Y_{a}+Y_{b})}.$$

**Figure 3:**
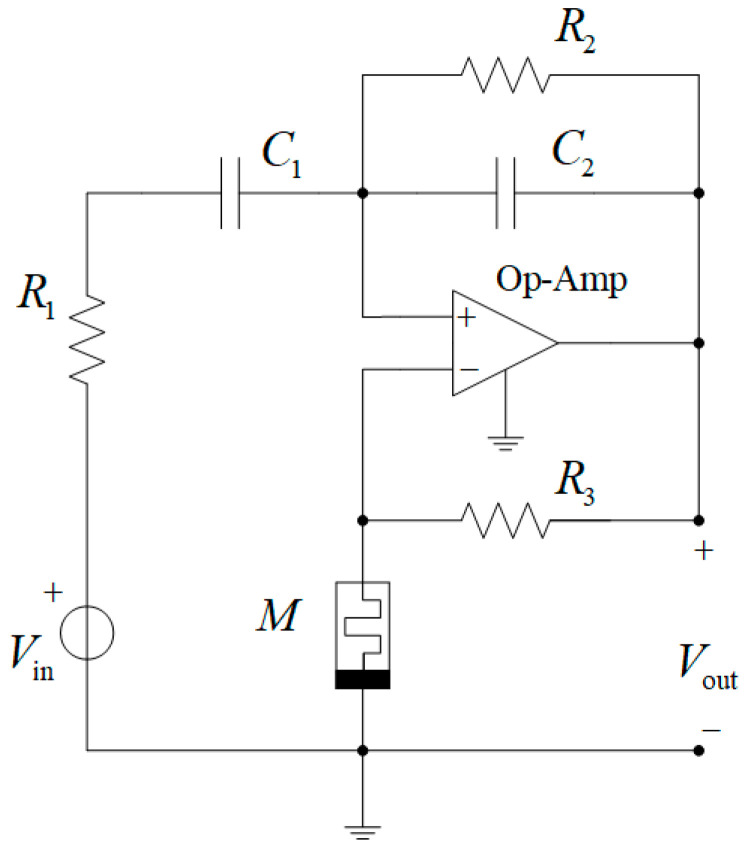
The schematic diagram of the memristive active bandpass Wien filter.

As an example, let us address the circuit of the active bandpass Wien filter with a memristor shown in Figure 3. Here, the bandpass filter is built on an operational amplifier, to which the inverting input of a Wien circuit is connected (two series-connected branches: one branch with series-connected elements $R_{1}$ and $C_{1}$, and the other branch with parallel-connected elements $R_{2}$ and $C_{2}$), and a voltage divider with a resistor $R_{3}$ and a memristor M is connected to the non-inverting input of the Op-Amp. The memristor is described by a mathematical model. Various types of mathematical memristor models are known, such as the linear ion drift model with different window functions, the nonlinear ion drift model, the Simmons tunnel barrier model, the TEAM model, the Yakopcic model, the VTEAM model, and others [48,49,50,51,52].

After comparing the circuits in Figure 1 and Figure 3, we get the following relationships:

(3) $$Y_{a}=G_{M}=1/M,\;Y_{b}=G_{3},\;Y_{c}=G_{2}+Y_{c2},\;Y_{d}=\frac{G_{1}Y_{c1}}{G_{1}+Y_{c1}},\;Y_{C1}=SC_{1},\;Y_{C2}=SC_{2},$$

where $G_{1}$, $G_{2}$, $G_{3}$ are conductances of the corresponding resistors; $C_{1}$, $C_{2}$ are capacitances of the corresponding capacitors; $G_{M}$ is the memdactance, equal to the reciprocal of the memristance M; and S is the variable of the Laplace transformation.

Firstly, we emphasize that variable M in Figure 3 can be described with any known mathematical model since M and general variable $Y_{a}$ (Equation (3)) are interrelated and then variable $Y_{a}$ is included in Equation (2) of the transfer function of the active Wien bridge circuit shown in Figure 1. Secondly, $R_{1}$, $R_{2}$, $R_{3}$, $C_{1}$, and $C_{2}$ are specified for simulation in the next part.

After substituting the variables from (3) into expression (2), we obtain the following transfer function of the Wien filter presented in Figure 3:

$$H(s)=\frac{G_{1}Y_{c1}}{G_{1}+Y_{c1}}(G_{3}+G_{M})\frac{1}{G_{3}\frac{(G_{2}+Y_{c2})(G_{1}+Y_{c1})+G_{1}Y_{c1}}{G_{1}+Y_{c1}}-(G_{2}+Y_{c2})(G_{3}+G_{M})}=$$

(4) $$=\frac{-S\frac{1}{R_{1}C_{2}}\left(m+1\right)}{S^{2}+\left[\frac{1}{R_{2}C_{2}}+\frac{1}{R_{1}C_{1}}-m\frac{1}{R_{1}C_{2}}\right]S+\frac{1}{R_{1}R_{2}C_{1}C_{2}}}=\frac{Sh_{0}}{S^{2}+S\frac{\omega_{0}}{Q}+\omega_{0}^{2}}$$

where $\omega_{0}$ is the angular resonant frequency; Q is the quality factor; m is the voltage divider coefficient (quality factor tuning coefficient), which is equal to

(5) $$m=\frac{M}{R_{3}}.$$

The advantage of the Wien filter is the ability to independently tune its frequency features, such as resonant frequency, quality factor, and gain at the resonant frequency. The specified features depend on the values of the filter components. Let us describe the derivation of these dependencies.

### 2.1. Dependence of the Resonant Frequency on the Wien Circuit Parameters

From the equivalence of denominators of the last two fractions in expression (4), we write the equalities

(6) $$\omega_{0}=\frac{1}{\sqrt{R_{1}R_{2}C_{1}C_{2}}},$$

and

(7) $$f_{0}=\frac{\omega_{0}}{2\pi }=\frac{1}{2\pi \sqrt{R_{1}R_{2}C_{1}C_{2}}},$$

where $f_{0}$ is the resonant frequency.

Under the equalities

(8) $$R_{1}=R_{2}=R,C_{1}=C_{2}=C,$$

frequencies in expressions (6) and (7) are written as

(9) $$\omega_{0}=\frac{1}{RC}\;\mathrm{and}\;f_{0}=\frac{1}{2\pi RC},$$

### 2.2. Dependence of the Quality Factor on the Voltage Divider Parameters at Given Parameters of the Wien Circuit

Based on the equivalence of denominators of the last two fractions in expression (4), we write the equality

$$\frac{\omega_{0}}{Q}=\frac{1}{R_{2}C_{2}}+\frac{1}{R_{1}C_{1}}-m\frac{1}{R_{1}C_{2}},$$

which, in view of expression (6), is rearranged as

$$\frac{1}{\sqrt{R_{1}R_{2}C_{1}C_{2}}}\frac{1}{Q}=\frac{C_{1}+C_{2}-mC_{2}}{R_{1}C_{1}C_{2}},$$

$$Q=\frac{1}{\sqrt{R_{1}R_{2}C_{1}C_{2}}}\frac{R_{1}C_{1}C_{2}}{C_{1}+C_{2}-mC_{2}}.$$

Under condition (8), the last expression can be written as

(10) $$Q=\frac{1}{2-m}.$$

Further, based on expression (5), we obtain the mathematical relationship between the memristance and the filter quality factor:

(11) $$M=R_{3}\left(2-\frac{1}{Q}\right).$$

The quality factor is known to be given by [3]

(12) $$Q=\frac{f_{0}}{\Delta f},$$

where $f_{0}$ is the resonant frequency; and Δf is the filter bandwidth determined according to the amplitude–frequency characteristic of the device.

In view of equality (12), we rewrite expression (11) as

(13) $$M=R_{3}\left(2-\frac{\Delta f}{f_{0}}\right).$$

Note that $f_{0}$ and Δf can be determined in the LTspice simulator.

### 2.3. Dependence of the Filter’s Gain at the Resonant Frequency on the Voltage Divider Parameters Under Given Parameters of the Wien Circuit

From the last equality of the fractions in expression (4) we get the expression

(14) $$h_{0}=-\frac{1}{R_{1}C_{2}}\left(m+1\right).$$

The last fraction in expression (4) describes the complex filter function as follows:

$$H(j\omega )=\frac{j\omega h_{0}}{-\omega^{2}+j\omega \frac{\omega_{0}}{Q}+\omega_{0}^{2}}$$

When substituting $\omega =\omega_{0}$ into it, we obtain

(15) $$H(j\omega_{0})=\frac{h_{0}Q}{\omega_{0}}.$$

In view of expressions (14), (10), and (6), we transform (15) into

$$H(j\omega_{0})=-\frac{1}{R_{1}C_{2}}\frac{(m+1)\sqrt{R_{1}R_{2}C_{1}C_{2}}}{\left(2-m\right)}.$$

Under condition (8), this equation can be written as

$$H(j\omega_{0})=-\frac{\left(m+1\right)}{\left(2-m\right)}.$$

Consequently, the maximum value (gain) $A_{\max}$ of the amplitude–frequency characteristic, achieved at the resonant frequency $\omega_{0}$, is equal to

(16) $$A_{\max}=\frac{m+1}{2-m}.$$

Taking into account Equation (5), we transform expression (16) to the form

(17) $$A_{\max}=\frac{M/R_{3}+1}{2-M/R_{3}}$$

and to the equation

$$m=\frac{2A_{\max}-1}{A_{\max}+1}.$$

Then, from expression (17), the memristance is equal to

(18) $$M=R_{3}\frac{2A_{\max}-1}{A_{\max}+1}.$$

Therefore, based on expressions (11), (13), and (18) at the given resonant frequency, we obtained the relationship between the memristance and the quality factor, bandwidth, and gain at the resonant frequency. Considering the resonant frequency $f_{0}$ to be dependent only on parameters of the Wien circuit ($R_{1},R_{2},C_{1},C_{2}$ in expression (6)) at constant resistance $R_{3}$, it follows from expression (13) that the smaller the memristance *M*, the wider the bandwidth Δf, and lower the quality factor Q and lower the gain $A_{\max}$ at the resonant frequency.

Let us consider the results of the analysis based on the transfer function (4) and circuit simulation (in LTspice [53,54,55]) of the memristive bandpass Wien filter, the circuit diagram of which is shown in Figure 3.

## 3. Results of Analysis and Simulation of the Active Memristive Wien Filter

**Figure 4:**
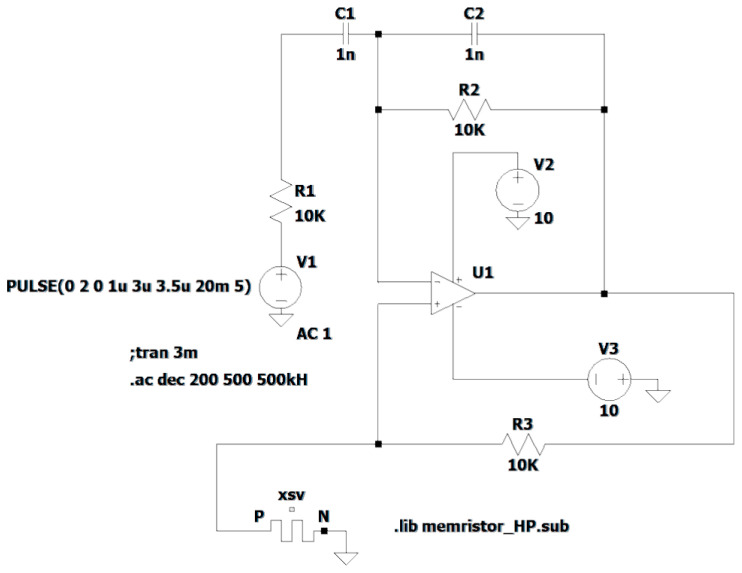
The circuit of the memristive Wien filter in LTspice.

The circuit of the memristive Wien filter, shown in Figure 3, is represented in the LTspice simulator as the circuit in Figure 4. The circuit parameters are as follows: $R_{1}=R_{2}=10\cdot 10^{3}\Omega$; $R_{3}=10\cdot 10^{3}\Omega$; $C_{1}=C_{2}=1\cdot 10^{-9}F$; U1 is an ideal Op-Amp; XSV is a memristor; *V*_1_ is the input voltage source; and $V_{2}=10V$ and $V_{3}=10V$ are the DC voltage sources for powering the Op-Amp.

The voltage divider connected to the positive input of the Op-Amp includes a memristor described by the linear ion drift model (Hewlett-Packard model, HP model) [48,49,50,51,52,56]. This model consists of two equations:

(19) $$v(t)=\left(R_{ON}x(t)+R_{OFF}\left(1-x(t)\right)\right)i(t),$$

(20) $$\frac{dx(t)}{dt}=\mu_{v}\frac{R_{ON}}{D^{2}}F(x)i(t),$$

where v(t), i(t), and x(t) are the voltage, current, and a normalized state variable of the memristor, respectively; $\mu_{v}$ is a constant representing the average ion mobility (memristor inertia); *D* is the thickness of the semiconductor film in the memristor; and F(x) is the window function.

The linear ion drift model is the simplest one among memristor models. Moreover, it can be flexible due to different window functions. This model describes physical memristors, the first of which was proposed by scientists from the Hewlett–Packard laboratory in 2008 [48,49,50,51,52]. This memristor consists of two metal electrodes, between which there is a width *D* insulator (the semiconductor film). Under the voltage impact, ions move from the doped insulator region with the lower resistance *R*_ON_ to the undoped insulator region with the higher resistance *R*_OFF_ and vice versa. The values of the model parameters correspond to physical memristors [51].

**Figure 5:**
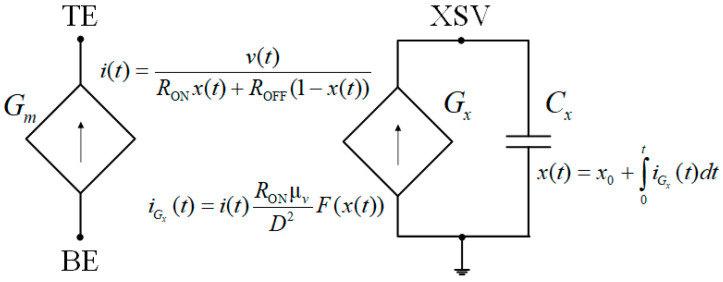
The circuit of the HP memristor model.

Based on the described mathematical model of the memristor, the schematic circuit shown in Figure 5 [57,58,59] is constructed. Expression (19) describes the left part of the circuit. This part includes element $G_{m}$, which is a voltage-controlled current source dependent on the voltage x(t) across the element $G_{x}$ (the voltage x(t) is also the voltage across the element $C_{x}$) in the right part of the circuit. The variables i(t) and v(t) are the current and voltage of the element $G_{m}$. The right part of the circuit in Figure 5 is constructed according to Equation (20). This part includes the element $G_{x}$ (a current source dependent on current i(t)), and the capacitor $C_{x}$, $C_{x}=1$ F. The variables $i_{G_{x}}(t)$ and x(t) are the current and voltage of both the element $G_{x}$ and the element $C_{x}$. The variable $x_{0}$ is the initial voltage across the capacitor $C_{x}$ from the expression

$$x(t)=x_{0}+\int_{0}^{t}i_{G_{x}}(t)dt.$$

The variable $x_{0}$ affects the memristance, and it adjusts the memristance. The circuit shown in Figure 5 is used to model the memristor in LTspice.

Let us present the LTspice netlist, which describes the circuit implementation (Figure 5) of the HP memristor model (Equations (19) and (20)) with the window function $F(x)=\delta_{1}(t)$, where $\delta_{1}(t)$ is the unit step function:

.params Ron = 100 Roff = 20 k x0 = 0.1 D = 10 n uv = 10 f

* Memristor I–V Relationship

.func IVRel(V1,V2) = V1/(Ron*V2 + Roff*(1−V2))

* Circuit to determine state variable

Gx 0 XSV value = {I(Gmem)*Ron*uv/pow(D,2)}

Cx XSV 0 {1}

.ic V(XSV) = x0

* Current source representing memristor

Gmem TE BE value = {IVRel(V(TE,BE),V(XSV,0))}

The analysis and simulation results of the memristive bandpass Wien filter in Figure 4 are presented in Table 1. The central column of Table 1 shows the values obtained at different $x_{0}$ through calculation of the filter’s frequency characteristics in LTspice: $f_{0,p}$ is a resonant frequency; Δf is a bandwidth; $Q_{p}$ is a quality factor, defined as $Q_{p}=\frac{f_{0,p}}{\Delta f};$ $M_{p}$ is the memristance, calculated from memristor currents and voltages obtained in the LTspice system; and $A_{\max,p}$ is the maximum value of the amplitude–frequency characteristic. The rightmost column of Table 1 contains the values $f_{0}$, Q, M, and $A_{\max}$, found from Equations (9), (12), (13), and (17), respectively.

**Figure 6:**
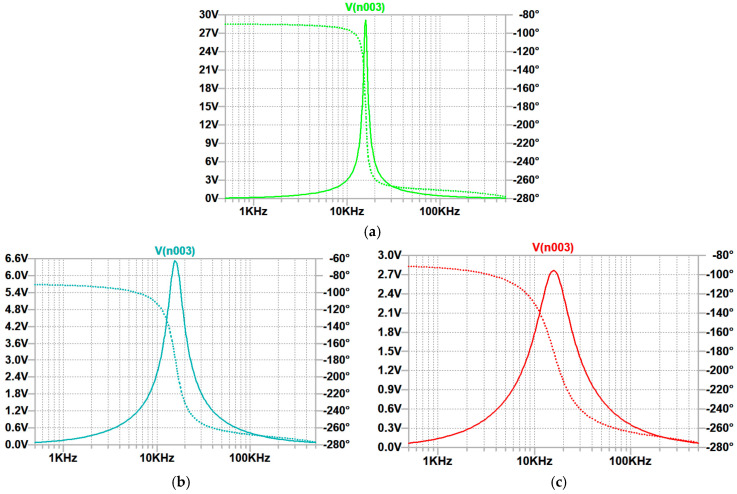
Amplitude–frequency and phase–frequency characteristics of the Wien filter with the HP memristor model: (**a**) for $x_{0}=0.05$, (**b**) for $x_{0}=0.2$, and (**c**) for $x_{0}=0.4$.

Figure 6 illustrates amplitude–frequency and phase–frequency characteristics of the Wien filter with a memristor, in the model of which different initial voltages (0.05; 0.2; 0.4) are applied across the capacitor $C_{x}$.

Table 1 and Figure 6 indicate that higher voltage $x_{0}$ means smaller memristance *M*, wider bandwidth Δf, lower quality factor Q, and lower gain Q at the resonant frequency. As follows from the analysis of Table 1, features $Q_{p}$, $M_{p}$, and $A_{\max,p}$, obtained through the circuit simulation of the memristive bandpass Wien filter (Figure 4) in the frequency domain using the LTspice simulator, and Q, M, and $A_{\max}$, found through the analytical modeling described in Section 2, are practically identical. The difference is less than 0.8%. This difference is reached under the harmonic input signal with amplitude equal to 1 and frequency within the bandwidth mentioned in Table 1. Thus, the analytical modeling and circuit simulation of the memristive bandpass Wien filter with an ideal Op-Amp have been correctly performed. This demonstrates that traditional methods of analysis and modeling of electrical circuits are applicable to memristive devices in view of mathematical memristor models.

## 4. Conclusions

The use of memristors provides new opportunities for high-quality tuning of parameters and characteristics of electronic devices. Being a passive electrical element, the memristor, with its advantages such as multilevel memristance, programmability for adjusting the memristance, high switching speed, nanoscale size, and low power consumption, ensures efficient tuning of the device parameters and characteristics. These advantages make the use of memristors as potentiometers preferrable compared to digital potentiometers.

This paper considers the simulation of the active bandpass Wien bridge with a memristor in the voltage divider connected to the positive input of the Op-Amp. The simulation was performed in the LTspice simulator. The HP model was used as a memristor model. Dependence of the resonant frequency on the Wien circuit parameters, dependence of the quality factor, and dependence of the filter’s gain at the resonant frequency on the voltage divider parameters for given parameters of the Wien circuit were obtained. Thus, independent tuning of the main frequency features of the Wien filter (gain, quality factor, and resonant frequency) has been demonstrated. Frequency features of the Wien filter were received both through analytical calculations and circuit simulation in the LTspice system. The difference between the obtained frequency features was less than 1%, confirming high accuracy of the performed simulation of the memristive Wien filter and the precision of its parameter tuning. Real electrical filters with implemented elements have potential challenges (noise impact, instability, and model mismatch). To solve these challenges, filters are built more constructively on the basis of memristors due to their advantages over digital potentiometers. Independent tuning of the main frequency parameters of the Wien filter helps to overcome differences between a physical filter and its model.

## Figures and Tables

**Table 1:** Values obtained from analysis and simulation of the active memristive bandpass Wien filter.

Initial Voltage $x_{0}$ Across $C_{x}$	Values Obtained from Frequency Responses in LTspice					Values Obtained from the Filter Transfer Function			
	$f_{0,p}$, kHz	Δf, kHz	$Q_{p}$	$M_{p}$, kΩ	$A_{\max,p}$	$f_{0}$, kHz	Q	M, kΩ	$A_{\max}$
0.05	15.82	1.56	10.14	19.01	29.08	15.91	10.20	19.01	29.30
0.1	15.83	3.14	5.04	18.01	14.06	15.91	5.06	18.01	14.07
0.2	15.91	6.3	2.52	16.02	6.53	15.91	2.52	16.04	6.57
0.3	15.92	9.43	1.69	14.03	4.02	15.91	1.69	14.07	4.06
0.4	15.91	12.5	1.27	12.04	2.77	15.91	1.27	12.14	2.82

## Data Availability

Data are contained within the article.

## References

[1] Izadian A. (2023). Fundamentals of Modern Electric Circuit Analysis and Filter Synthesis: A Transfer Function Approach.

[2] Schubert T.F., Kim E.M. (2016). Fundamentals of Electronics: Book 3 Active Filters and Amplifier Frequency Response.

[3] Deliyannis T., Sun Y., Fidler J.K. (2019). Continuous-Time Active Filter Design.

[4] Wang X., Wang A., Wang D., Wang W. (2022). An Improved Spline Adaptive Filter for Nonlinear System Identification under Impulsive Noise Environment. Energy Rep..

[5] Masa A.P.A., Fajri M.M.H., Septiarini A., Winarno E. (2024). Comparison of Noise Using Reduction Method for Repairing Digital Image. JOIV Int. J. Inform. Vis..

[6] Solovyeva E.B. (2017). A Split Signal Polynomial as a Model of an Impulse Noise Filter for Speech Signal Recovery. J. Phys. Conf. Ser..

[7] Solovyeva E., Abdullah A. (2022). Dual Autoencoder Network with Separable Convolutional Layers for Denoising and Deblurring Images. J. Imaging.

[8] Solovyeva E.B. (2015). Cascade Structure of Digital Predistorter for Power Amplifier Linearization. Radioengineering.

[9] Solovyeva E. Operator Approach to Nonlinear Compensator Synthesys for Communication Systems. Proceedings of the 2016 International Siberian Conference on Control and Communications (SIBCON).

[10] Nitsch J., Solovyeva E., Korovkin N., Scheibe H.-J. Occurrence of Low-Frequency Noises in Electronic Systems under Action of Two-Tone High-Frequency Electromagnetic Excitation. Proceedings of the 2005 International Symposium on Electromagnetic Compatibility, EMC 2005.

[11] Abdelnaby M., Alnajjar R., Bensmida S., Hammi O. (2024). Reduced Complexity Sequential Digital Predistortion Technique for 5G Applications. Smart Cities.

[12] Li X. (2024). Simulation of Motion Nonlinear Error Compensation of CNC Machine Tools with Multi-Axis Linkage. Scalable Comput. Pract. Exp..

[13] Liu J., Xia Y., Liu G., Lyu L., Nie Y., Mei D., Chen Z. (2024). Motion Control of Electro-Hydrostatic Actuators With Modeling and Compensation of Nonlinear Bulk Modulus. IEEE Trans. Ind. Electron..

[14] Li S., Liu S., Wang J., Yan S., Liu J., Du Z. (2024). Adaptive-Wavelet-Threshold-Function-Based M2M Gaussian Noise Removal Method. IEEE Internet Things J..

[15] Kuş Z., Aydin M. Removal of Gaussian Distributed Noise in Images with Deep Neural Network Models. Proceedings of the 2022 30th Signal Processing and Communications Applications Conference (SIU).

[16] Vigneshwaran S., Santhoshkumar A., Srikanth S. (2016). Design and Analysis of Active High Pass, Low Pass & Band Pass Butterworth Filters Using LM741. Int. J. Eng. Sci. Comput..

[17] Severo L.C., Van Noije W. A 10.9-μW/Pole 0.4-V Active-RC Complex BPF for Bluetooth Low Energy RF Receivers. Proceedings of the 2018 IEEE 9th Latin American Symposium on Circuits & Systems (LASCAS).

[18] Agrawal D., Maheshwari S. (2021). High-Performance Electronically Tunable Analog Filter Using a Single EX-CCCII. Circuits Syst. Signal Process..

[19] Agrawal D., Tripathi S.K., Reddy N.S.S., Reddy M.S.V., Shoaib P.M. (2024). Design a Configurable First Order Universal Filter Using a Single EX-CCCII. Russ. Microelectron..

[20] Abd Algaffar A.N., Ali Jasem N., Ibrahim Abbo A. (2019). Notch Filters Design with Enhanced Performance. J. Phys. Conf. Ser..

[21] Winder S. (2002). Analog and Digital Filter Design.

[22] Solovyeva E.B., Inshakov Y.M., Ezerov K.S. Using the NI ELVIS II Complex for Improvement of Laboratory Course in Electrical Engineering. Proceedings of the 2018 IEEE International Conference “Quality Management, Transport and Information Security, Information Technologies” (IT&QM&IS).

[23] Denisenko D.Y., Prokopenko N.N., Ivanov Y.I., Zhebrun E.A. Band-Pass ARC-Filter Based on the Classical Wien Bridge with the Pole Frequency Rise and Independent Adjustment of the Main Parameters. Proceedings of the 2018 IEEE International Conference on Electrical Engineering and Photonics (EExPolytech).

[24] Li Y.-A. (2023). Four Electronically Tunable Wien-Bridge Sinusoidal Oscillators. Proc. Natl. Acad. Sci. India Sect. A Phys. Sci..

[25] Elwy O., Said L.A., Madian A.H., Radwan A.G. (2019). All Possible Topologies of the Fractional-Order Wien Oscillator Family Using Different Approximation Techniques. Circuits Syst. Signal Process..

[26] Bao H., Wang N., Wu H., Song Z., Bao B. (2019). Bi-Stability in an Improved Memristor-Based Third-Order Wien-Bridge Oscillator. IETE Tech. Rev..

[27] Ndassi H.L., Tchendjeu A.E.T., Motchongom Tingue M., Kengne E.R.M., Tchitnga R., Tchoffo M. (2020). Complex Dynamics of a Modified Four Order Wien-Bridge Oscillator Model and FPGA Implementation. Eur. Phys. J. Plus.

[28] Komanapalli G., Pandey R., Pandey N. (2019). Operational Transresistance Amplifier Based Wienbridge Oscillator and Its Harmonic Analysis. Wirel. Pers. Commun..

[29] Mahdavi M. Reconfigurable Band-Stop Filter Design Using Computing In Memory. Proceedings of the 2024 IEEE International Conference on Communications, Control, and Computing Technologies for Smart Grids (SmartGridComm).

[30] Lindberg E. The Wien Bridge Oscillator Family. Proceedings of the ICSES-06.

[31] Ahmed H.B., Saleh A.H., Humood K.A., Mahmood T. (2024). Design Wien Bridge Oscillator for VLF to VHF Using Practical Op—Amp. Int. J. Electr. Electron. Res..

[32] Abuelma’atti M.T., Khalifa Z.J. A Memristor Based Wien-Bridge Sinusoidal/Chaotic Oscillator. Proceedings of the 2016 International Conference on Electronics, Information, and Communications (ICEIC).

[33] Pershin Y.V., Di Ventra M. (2010). Practical Approach to Programmable Analog Circuits With Memristors. IEEE Trans. Circuits Syst. I Regul. Pap..

[34] Shin S., Kim K., Kang S.-M. (2011). Memristor Applications for Programmable Analog ICs. IEEE Trans. Nanotechnol..

[35] Tan J., Duan S., Yang T., Zhu H., Cong F., Leung A., Wei Q. (2017). A Programmable Memristor Potentiometer and Its Application in the Filter Circuit. Proceedings of the Advances in Neural Networks—ISNN 2017.

[36] Pandiev I.M. (2018). Analysis and Behavioral Modeling of Monolithic Digital Potentiometers. IEEE Trans. Ind. Appl..

[37] Tu S., Li J., Ren Y., Jiang Q., Xiong S. (2023). A Novel Programming Circuit for Memristors. Microelectron. Eng..

[38] Isah A., Bilbault J.-M. (2022). Review on the Basic Circuit Elements and Memristor Interpretation: Analysis, Technology and Applications. J. Low Power Electron. Appl..

[39] De Souza Dias C., Butzen P.F. (2021). Memristors: A Journey from Material Engineering to beyond von-Neumann Computing. J. Integr. Circuits Syst..

[40] Im I.H., Kim S.J., Jang H.W. (2020). Memristive Devices for New Computing Paradigms. Adv. Intell. Syst..

[41] Thakkar P., Gosai J., Gogoi H.J., Solanki A. (2024). From Fundamentals to Frontiers: A Review of Memristor Mechanisms, Modeling and Emerging Applications. J. Mater. Chem. C.

[42] Pazos S., Xu X., Guo T., Zhu K., Alshareef H.N., Lanza M. (2024). Solution-Processed Memristors: Performance and Reliability. Nat. Rev. Mater..

[43] Udaya Mohanan K. (2024). Resistive Switching Devices for Neuromorphic Computing: From Foundations to Chip Level Innovations. Nanomaterials.

[44] Barraj I., Mestiri H., Masmoudi M. (2024). Overview of Memristor-Based Design for Analog Applications. Micromachines.

[45] Yang X., Taylor B., Wu A., Chen Y., Chua L.O. (2022). Research Progress on Memristor: From Synapses to Computing Systems. IEEE Trans. Circuits Syst. I Regul. Pap..

[46] Lv Z., Jiang M., Liu H., Li Q., Xie T., Yang J., Wang Y., Zhai Y., Ding G., Zhu S. (2025). Temperature-Resilient Polymeric Memristors for Effective Deblurring in Static and Dynamic Imaging. Adv. Funct. Mater..

[47] Lv Z., Zhu S., Wang Y., Ren Y., Luo M., Wang H., Zhang G., Zhai Y., Zhao S., Zhou Y. (2024). Development of Bio-Voltage Operated Humidity-Sensory Neurons Comprising Self-Assembled Peptide Memristors. Adv. Mater..

[48] Mladenov V. (2023). Application and Analysis of Modified Metal-Oxide Memristor Models in Electronic Devices. Technologies.

[49] Mladenov V., Kirilov S., Zaykov I. A General Model for Metal Oxide-Based Memristors and Application in Filters. Proceedings of the 2022 11th International Conference on Modern Circuits and Systems Technologies (MOCAST).

[50] Oğuz Y., James A.P. (2018). Mathematical Modeling of Memristors. Memristor and Memristive Neural Networks.

[51] Maruf M.H., Ali S.I. (2020). Review and Comparative Study of I-V Characteristics of Different Memristor Models with Sinusoidal Input. Int. J. Electron..

[52] Mladenov V.M., Zaykov I.D., Kirilov S.M. Application of a Nonlinear Drift Memristor Model in Analogue Reconfigurable Devices. Proceedings of the 2022 26th International Conference Electronics.

[53] Mladenov V. (2021). A Unified and Open LTSPICE Memristor Model Library. Electronics.

[54] Dautovic S., Samardzic N., Juhas A., Ascoli A., Tetzlaff R. (2024). Analytical Solutions for Charge and Flux in HP Ideal Generic Memristor Model With Joglekar and Prodromakis Window Functions. IEEE Access.

[55] Amisha A.A., Pravin U.D., Sanjay M.H., Kirti S.A. (2014). Implementation of Memristor Circuits Using LTspice. Int. Res. J. Manag. Sci. Technol..

[56] Mladenov V.M., Kirilov S.M. A Simple Memristor Model for Memory Crossbars. Proceedings of the 2024 12th International Scientific Conference on Computer Science (COMSCI).

[57] Biolek Z., Biolek D., Biolkova V. (2009). SPICE Model of Memristor with Nonlinear Dopant Drift. Radioengineering.

[58] Fatima M., Begum R. (2018). Power Dissipation Analysis of Memristor for Low Power Integrated Circuit Applications. Int. J. Sci. Res. Sci. Eng. Technol..

[59] Ahmed Z.K., Taha F.H. (2021). Evaluation and Realization of Memristor Emulator Spice. J. Phys. Conf. Ser..

